# Genome-wide analysis and characterization of F-box gene family in *Gossypium hirsutum* L

**DOI:** 10.1186/s12864-019-6280-2

**Published:** 2019-12-19

**Authors:** Shulin Zhang, Zailong Tian, Haipeng Li, Yutao Guo, Yanqi Zhang, Jeremy A. Roberts, Xuebin Zhang, Yuchen Miao

**Affiliations:** 1 State Key Laboratory of Cotton Biology, Institute of Plant Stress Biology, School of Life Sciences, Henan University, Jinming Street, Kaifeng, 475004 China; 2 College of Biology and Food Engineering, Anyang Institute of Technology, Anyang, 455000 China; 3 Faculty of Science and Engineering, School of Biological & Marine Sciences, University of Plymouth, Devon, UK

**Keywords:** *Gossypium hirsutum* L., Cotton, F-box gene family, Ubiquitination, Protein degradation

## Abstract

**Background:**

F-box proteins are substrate-recognition components of the Skp1-Rbx1-Cul1-F-box protein (SCF) ubiquitin ligases. By selectively targeting the key regulatory proteins or enzymes for ubiquitination and 26S proteasome mediated degradation, F-box proteins play diverse roles in plant growth/development and in the responses of plants to both environmental and endogenous signals. Studies of F-box proteins from the model plant *Arabidopsis* and from many additional plant species have demonstrated that they belong to a super gene family, and function across almost all aspects of the plant life cycle. However, systematic exploration of F-box family genes in the important fiber crop cotton (*Gossypium hirsutum*) has not been previously performed. The genome-wide analysis of the cotton F-box gene family is now possible thanks to the completion of several cotton genome sequencing projects.

**Results:**

In current study, we first conducted a genome-wide investigation of cotton F-box family genes by reference to the published F-box protein sequences from other plant species. 592 F-box protein encoding genes were identified in the *Gossypium hirsutume* acc.TM-1 genome and, subsequently, we were able to present their gene structures, chromosomal locations, syntenic relationships with their parent species. In addition, duplication modes analysis showed that cotton F-box genes were distributed to 26 chromosomes, with the maximum number of genes being detected on chromosome 5. Although the WGD (whole-genome duplication) mode seems play a dominant role during cotton F-box gene expansion process, other duplication modes including TD (tandem duplication), PD (proximal duplication), and TRD (transposed duplication) also contribute significantly to the evolutionary expansion of cotton F-box genes. Collectively, these bioinformatic analysis suggest possible evolutionary forces underlying F-box gene diversification. Additionally, we also conducted analyses of gene ontology, and expression profiles in silico, allowing identification of F-box gene members potentially involved in hormone signal transduction.

**Conclusion:**

The results of this study provide first insights into the *Gossypium hirsutum* F-box gene family, which lays the foundation for future studies of functionality, particularly those involving F-box protein family members that play a role in hormone signal transduction.

## Background

The Ubiquitin (Ub)/26S proteasome pathway is an important post-translational regulatory process in eukaryotes that marks unwanted or misfolded proteins for degradation. This pathway also serves to adjust the activities of key regulatory proteins, and such processes being used by cells to respond rapidly to intracellular signals and environmental stimuli [1, 2]. Ubiquitination of target proteins occurs in the Ub/26S proteasome pathway predominantly via three enzymatic reactions. First, an ATP-dependent activation of ubiquitin is catalyzed by enzyme E1, then the activated ubiquitin is transferred to the ubiquitin-conjugating enzyme E2, and, finally, the ubiquitin is selectively bound to substrate proteins directed by the ubiquitin-protein ligase E3. The E3 ligase in the Ub/26S proteasome pathway is essential for recognition of target proteins for ubiquitination, and is the specificity determinant of the E3 complex for appropriate targets [3]. To date, several hundred E3 ubiquitin ligases have been identified, one of the best characterized being the SCF protein complex consisting of RBX1, SKP1, CULLIN, and F-box proteins [4, 5]. In this complex, RBX1, CULLIN1, and SKP1 are invariant, and interact together to form a core scaffold. SKP1 further interacts with a specific F-box protein. F-box proteins found within the SCF complexes vary significantly in sequence. As the name suggests, proteins in this family contain at least one conserved F-box motif of 40–50 amino acids at their N-terminus which interacts with the SKP1 protein. In contrast, the C-terminal region of F-box proteins usually contain highly variable protein-protein interaction domains which serve to specifically recruit substrate proteins for ubiquitination and subsequent 26S proteasome degradation. Therefore, F-box proteins play a crucial role for defining the specific substrates of the SCF complexes for destruction [6, 7].

As a result of rapid advances in DNA sequencing technologies, hundreds of F-box genes have been identified in the genome of every plant species sequenced, including *Arabidopsis* [8], rice [8], poplar [8], soybean [9], Medicago [10], maize [11], chickpea [12], apple [13] and pear [14], respectively containing 692, 779, 337, 509, 359, 285, 517, and 226 F-box genes. In addition to the N-terminus F-box domain, the variable protein-protein interaction motifs found at the C termini of F-box proteins can be used to classify F-box proteins into different subfamilies based on the presence of interaction motifs such as leucine-rich repeats (LRR), Kelch, WD-40, Armadillo (Arm), tetratricopeptide repeats (TPRs), Tub, actin, DEAD-like helicase, and jumonji (JmjC) [15]. The large number of F-box proteins theoretically forms a diverse array of SCF complexes which, in turn, will recognize a wide range of substrate proteins for ubiquitination and degradation. Functional characterization of a limited number of plant F-box genes have demonstrated that F-box proteins are associated with many important cellular processes such as embryogenesis [16, 17], seed germination [18], plant growth and development [19, 20], floral development [14, 21], responses to biotic and abiotic stress [22–24], plant secondary metabolism [25–27], hormonal responses, and senescence [4, 28, 29].

Worldwide, cotton is an extremely important fiber crop. Upland cotton (*Gossypium hirsutum*) is the primary cultivated species, contributing more than 90% of global cotton fiber production [30–32]. *Gossypium hirsutum* is also one of the descendant allotetraploid species and is believed to be derived from polyploidization between a spinnable-fiber-capable A genome species (*Gossypium arboreum*) and a non-spinnable-fiber-capable D genome species (*Gossypium raimondii*) [33]. Systematic exploration of F-box family genes in cotton (*Gossypium hirsutum*) had not been previously performed due to the incomplete state of cotton genome sequencing projects. Collectively, only a few F-box proteins have been functionally explored in *Gossypium hirsutum*, including two putative homologues of the *MAX2* genes that have been shown to control shoot lateral branching in *Arabidopsis* [34]. In a second study, Wei et al. [35] cloned a *GhFBO* (GenBank:JF498592) gene containing two Tubby C-terminal domains, and showed that this gene had elevated levels of expression in flower, stem, and leaf tissues. But the detailed biological function of *GhFBO* was not examined in their studies. With the completion of genome sequencing projects for an increasing number of cotton species, F-box protein encoding genes in *Gossypium hirsutum* have become amenable to a systematic investigation of their structures and syntenic relationships for further functionality studies.

In our current study, we present the results of a genome-wide analysis of F-box genes in *Gossypium hirsutum*. 592 F-box protein encoding genes were identified in the *Gossypium hirsutum*e acc.TM-1 genome, and their gene structures, chromosomal locations, syntenic relationships across other cotton species, and duplication modes are presented, along with a discussion of the possible evolutionary effects on allotetraploid cotton F-box genes. Finally, we investigated gene ontology, the expression profiles of all F-box based on publicly available databases and the possible F-box gene members involved in hormone signal transduction. Our results provide the first overview of the *Gossypium hirsutum* F-box gene family, which we believe will lay the foundation for future functionality studies, particularly the F-box proteins that likely play important roles in hormone signal transduction.

## Methods

### Identification and classification of F-box genes from *Gossypium hirsutum*

To identify the F-box proteins from *Gossypium hirsutum*, the local BLASTP algorithm (with an E-value cut off of 1e-10) was applied to the *Gossypium hirsutum* genome database (http://mascotton.njau.edu.cn) [36] in a global search for F-box proteins. The initial query sequences were the 1808 previously published F-box protein sequences from *Arabidopsis*, *Populus trichocarpa*, and rice [8]. After this initial screening, all F-box protein candidates were verified by the Pfam (http://pfam.sanger.ac.uk/search) and SMART (http://smart.embl-heidelberg.de) webserver, with an e-value cut-off of less than 1.0 to ensure each candidate sequence contained at least one of the F-box motifs (PF00646, PF12937, PF13013, PF04300, PF07734, PF07735, PF08268 and PF08387). All proteins containing these F-box domains were considered to be F-box proteins from *Gossypium hirsutum*. According to their C-terminal protein-protein interaction domains, the identified cotton F-box proteins were further classified into different subfamilies. In order to understand the evolution of the expansion of the cotton F-box genes, the F-box protein encoding genes from *Gossypium raimondii* and *Gossypium arboreum* were also identified and classified using the same approach.

### Dissection of different duplication modes of F-box genes from *Gossypium hirsutum*

The MCScanX-transposed software package [37] was used to predict the genomic duplication mode of *Gossypium hirsutum* F-box genes, based on syntenic analyses comparing allotetraploid and corresponding diploids. F-box genes within the *Gossypium hirsutum* genome were classified as transposed, proximal, tandem, or whole-genome duplications (WGD). First, the local BLASTP algorithm was used to compare *Gossypium hirsutum* versus *Gossypium hirsutum, Gossypium hirsutum* versus *Gossypium raimondii,* and *Gossypium hirsutum* versus *Gossypium arboretum*, for all F-box proteins from the AD, A2 and D5 genome (E < 1e-5, top five matches and m8 format) without the scaffold gene. Second, the core program of MCScanX-transpose was executed using the BLASTP output (*Gossypium hirsutum* versus *Gossypium raimondii,* and *Gossypium hirsutum* versus *Gossypium arboreum* as the outgroup) and the annotation file (.ggf file) as the input. Finally, syntenic colinear gene pairs between allotetraploid and diploids, and the F-box gene from *Gossypium hirsutum* duplication mode were produced.

### Calculation of nonsynonymous (*Ka*) and synonymous (*Ks*) substitution rates and Ka/Ks ratios

Verified duplicated gene pairs originating from different duplication modes were used to calculate the *Ka* and *Ks* substitution rates. First, the coding sequences of duplicated genes were compared by LASTZ -master tools (http://www.bx.psu.edu/~rsharris/lastz) and an AXT file was produced. Then KaKs_Calculator 2.0 was used to estimate *Ka* and *Ks* values, and the *Ka/Ks* ratios were calculated based on the AXT file with model-averaged method. The parameters were configured as described in the software package manuals [38, 39] . The Ka/Ks ratio was assessed to determine the molecular evolutionary rates of each gene pair. In general, Ka/Ks < 1 indicates purifying selection; Ka/Ks = 1 indicates neutral selection; and Ka/Ks > 1 indicates positive selection. The divergence time of these gene pairs was estimated using the formula “t = Ks/2r”, with r (2.6 × 10^− 9^) representing neutral substitution [36, 40].

### Gene ontology (GO) items and expression pattern analysis

The GO annotation for cotton F-box protein encoding genes was obtained from the *Gossypium hirsutum* L. acc. TM-1 genome project [36]. The three top GO categories: molecular function (MF), biological process (BP), and cellular component (CP) were analyzed. The functional annotations of F-box genes involved in any biological process (BP) were predicted based on putative homologues from *Arabidopsis. thaliana*. Expression data for all F-box protein-encoding genes were obtained from CottonFGD (https://cottonfgd.org/analyze) for 9 tissues (Calycle, Leaf, Petal, Pistil, Root, Stamen, Stem, Torus, fiber). The log2 transformed RPKM (reads per kilobase per million) values or TPM (transcripts copies per million tags) values were used to measure expression levels of the F-box genes, and to generate heat maps. Expression clusters were defined using Mev4.6.2 software (http://www.tm4.org/mev.html).

For in silico expression analyses, RNA-seq data for 8 *Gossypium hirsutum* L. acc. TM-1 tissues (torus, stem, leaf, root, 5dap fiber, 10dap fiber,15dap fiber and 25dap fiber) were downloaded from the NCBI SRA database (SRA available accession numbers SRX797899, SRX797900, SRX79901, SRX797902, SRX797917, SRX797918, SRX797919 and SRX797920 respectively [36]). All analyses were carried out using the Tophat-Cufflinks pipeline, with the following versions: Bowtie2 v2.3.4.3, Tophat v2. 1.1, Samtools v1.9 and Cufflinks v2.2.1. The *G. hirsutum* acc.TM-1 genome and gene model annotation file (GFF, gene. Ghir.NAU.gff3) downloaded from cotton gene (https://www.cottongen.org/) were used as reference. The FPKM values for F-box genes were utilized for K-means clustering using the XLSTAT version 2013 and standardized for generating the heatmaps using R software.

### Identification of F-box gene as the SCF complexes involved in hormone signal transduction pathway

To identify the *Gossypium hirsutum* F-box genes which can potentially form the SCF complexes involved in plant hormone signal transduction pathways, we first obtained the protein sequences of the *Arabidopsis* F-box proteins involved in hormone signal transduction based on previous studies, including *TIR1* in the auxin signaling pathway, *SLY1* in the gibberellin signaling pathway, *EBF2* in the ethylene signaling pathway and the F-box genes that have been proposed to play a role in the ABA signaling pathway [41, 42]. Second, we performed a local BLASTP algorithm-based search (E < 1e-10 and Identities > 50%) against all F-box protein sequences using the above listed protein sequences from *Arabidopsis* as queries. From these results, a number of candidate F-box genes likely involved in cotton IAA, JA, GA, ABA and ethylene signal transduction pathways were chosen, and their expression responses to different hormone treatments determined by qRT-PCR.

### RNA extraction and qRT-PCR

To examine expression profiles of F-box protein encoding genes in hormone signal transduction pathways, *Gossypium hirsutum* L. acc. TM-1 leaves at the four-leaf stage were submerged in 100 μM ABA (Biotopped, cat number: A1049) solution, 100 μM ACC (Ruitaibio) solution, and 100 μM GA3 (Biotopped) solution, or were sprayed with 100 μM IBA solution (Solarbio, cat number: 531A0214), respectively. Samples were collected from leaves at 0, 1, 3, 6, and 12 h after treatment. Samples collected at 0 h were used as controls. All samples were immediately frozen in liquid nitrogen and kept at − 80 °C proir to total RNA extraction. Total RNA was extracted from the samples using the RNAprep Pure Kit (For Plants) (TIANGEN, Beijing, China). First-strand cDNA was synthesized based on reverse transcription of 1 μg RNA digested by DNase I using the PrimeScript™ RT Reagent Kit (Takara, Dalian, China). PCR amplifications were performed using SYBR® Premix Ex Taq™ (Takara). For real-time PCR, gene-specific primers were designed using Primer 5.0 (Additional file 5: Table S8). For the qRT-PCR assay, cDNA was diluted to 100 ng/μL with ddH_2_O. The reaction (in a total volume of 20 μL) contains 10 μL SYBR® Premix Ex Taq™ (2×), 0.4 μL of each primer (10 μM), 0.4 μl ROX Reference Dye (50×), 1 μL template (about 100 ng/μL), and ddH_2_O to make up the total volume. The qRT-PCR reaction was performed on a ROCHE Real-time PCR System (Applied Biosystems) as described [43]. Fold-changes were calculated using the comparative CT method (2-ΔΔCt), using cotton GhActin1 as an internal reference [44].

## Results

### Identification and classification of F-box genes in *Gossypium hirsutum*

A total of 30,687 F-box encoding sequences were initially identified by local BLASTP. After the repetitive sequences were removed, 2904 sequences were retained, and were submitted to the Pfam and SMART webserver to confirm that the identified F-box proteins contained at least one of the established F-box domains. After this step, 592 cDNAs were ultimately verified as *Gossypium hirsutum* F-box genes, and were named based on their chromosomal locations. Gene names, IDs, chromosomal locations, exon numbers, amino acid composition, molecular weights and pIs are listed in Additional file 5: Table S1. In addition, 300 F-box genes from *Gossypium raimondii* and 282 F-box genes from *Gossypium arboreum* were also separately identified using the same approaches (Additional file 5: Table S2 and Table S3). According to cotton origin and evolution studies [30–32, 45], the domesticated *Gossypium hirsutum* (allotetraploid AD-hybrid) species are the offspring formed between diploid cotton species *Gossypium raimondii* (D-genome) and *Gossypium arboreum* (A-genome). The polyploidization between the A-genome and D-genome species leads to the tetraploid AD species containing two copies of the entire A and D genomes, which instead of two copies of each genome (one from each parent), has four (two from each parent). Interestingly, the AD offspring are quite different from both the parents in terms of fiber qualities, and stress and disease resistance, indicating that the AD genome rearrangements/combinations have caused not only the genome size doubling but also potential gene expression changes. In our current studies, we found that *Gossypium hirsutum* possesses almost twice the number of F-box genes as compared to its diploid parents *Gossypium arboretum* and *Gossypium raimondii*, which indicates that most of the F-box genes are retained after polyploidization between the two diploid cotton species, *Gossypium raimondii* and *Gossypium arboreum.*

**Fig. 1:**
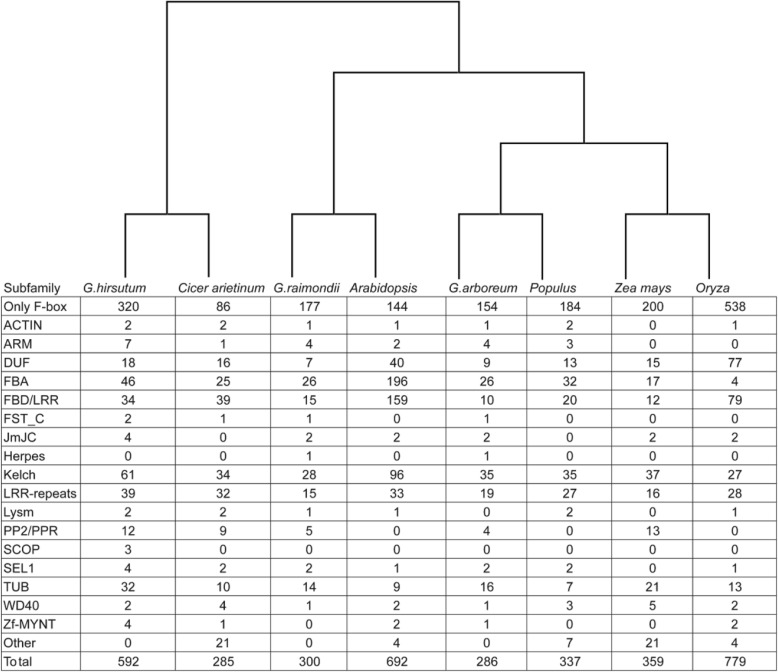
The number and classicization of F-box genes identified in *G. hirsutum*, *G.Raimondi* and *G.arboreum* genomes. All the F-box genes were classified into different subfamilies based on their C-terminus functional domains (Pfam domains)

According to the functional domains found within the C-terminal region of the identified cotton F-box proteins, they can be grouped into 17 different subfamilies (Fig. 1). The F-box protein subfamily containing no-known C-terminal functional domains, designated as Fbox, is the largest cotton F-box gene subfamily containing 320 members. The remaining F-box proteins were divided into 16 subfamilies according to the presence of well-defined C-terminal functional domains, such as Actin (2 genes), ARM (7 genes), DUF (18 genes), FBA (46 genes), FBD/LRR (34 genes), FST_C (2 genes), JmJC (4 genes), Kelch (61 genes), LRR-Repeat (39 genes), Lysm (2 genes), PP2/PPR (12 genes), SCOP (3 genes), SEL1(4 genes), Tub (32 genes), WD40 (2 genes), and zf-MYNT (4 genes) (Fig. 1). It is interesting that, based on the Pfam database, the SCOP subfamily is present only in *Gossypium hirsutum*, and that the Herpes subfamily is absent in *Gossypium hirsutum* when compared with the F-box protein subfamilies in *Gossypium raimondii* and *Gossypium arboreum*. Three genes in the *Gossypium hirsutum* SCOP subfamily contain the cullin domain (PF00888) which usually are not present in plant F-box proteins. Cullin proteins, which are conserved in all eukaryotes, normally play roles as scaffold proteins supporting other components of the E3 ubiquitin ligase complexes. In the SCF complex, Cullin proteins usually link F-box proteins with the remaining members of SCF complexes, which likely allows the cotton SCOP F-box subfamily proteins to recruit their substrate proteins independently from the SCF complexes. In addition, the Herpes subfamily (Herpes_UL92(PF03048)) was only found in *Gossypium raimondii* and *Gossypium arboreum*, and not in *Gossypium hirsutum*, suggesting that *Gossypium hirsutum* experienced different forces of selection during cotton polyploidization [46]. Chromosomal breakages and rearrangements leading to different patterns of gene loss and gene retention during the polyploidization represents a possible explanation for this phenomenon [47].

### The genomic distribution and gene expansion events of *Gossypium hirsutum* F-box genes

**Fig. 2:**
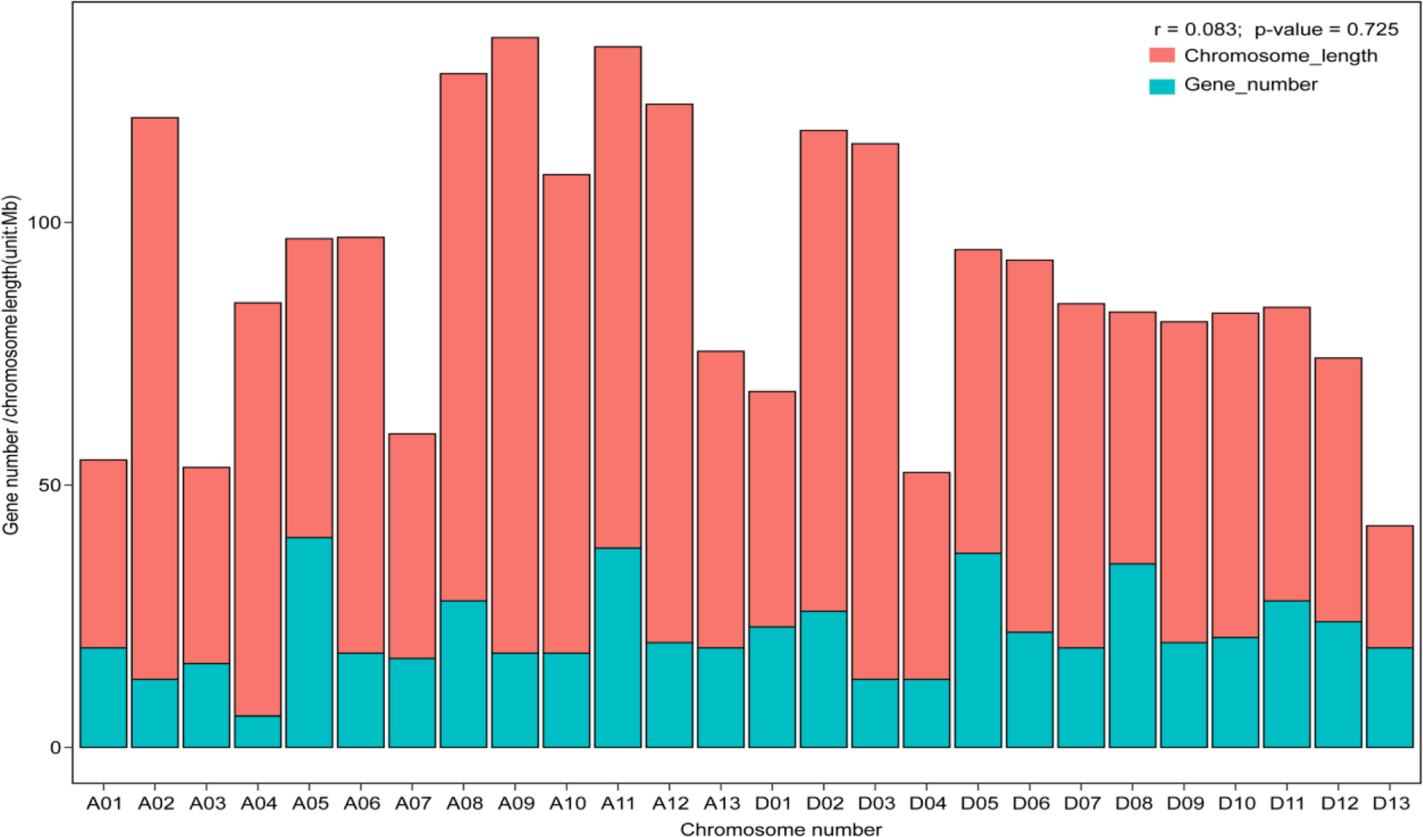
The distribution of F-box genes on the 26 *G. hirsutum* chromosomes. The correlation between number of F-box genes and chromosome length was evaluated by Pearson correlation coefficient (*r* = 0.083 *p*-value = 0.725)

Using the genome sequence of *Gossypium hirsutum* acc.TM-1 as a reference, the 592 F-box protein encoding genes were mapped to individual chromosomes or scaffolds. Of these, 524 F-box genes were assigned to 26 chromosomes, with the maximum number of genes being detected on chromosome 5 (37 genes), followed by chromosome 11 (36 genes), chromosome 18 (34 genes) and chromosome 21 (34 genes) respectively. Chromosome 4 contained the fewest F-box genes (6 genes), with the remaining 68 F-box genes being located on unmapped scaffolds. Notably, longer chromosomes do not necessarily contain more F-box gene family members, indicating that the number of F-box genes on each chromosome is not correlated to length (Pearson correlation r = 0.083 *p*-value = 0.725) (Fig. 2). This result demonstrates that cotton F-box protein encoding genes, like the F-box genes in other plant species, are unevenly distributed on the 26 chromosomes of *Gossypium hirsutum* [11, 12, 14, 15, 48].

**Fig. 3:**
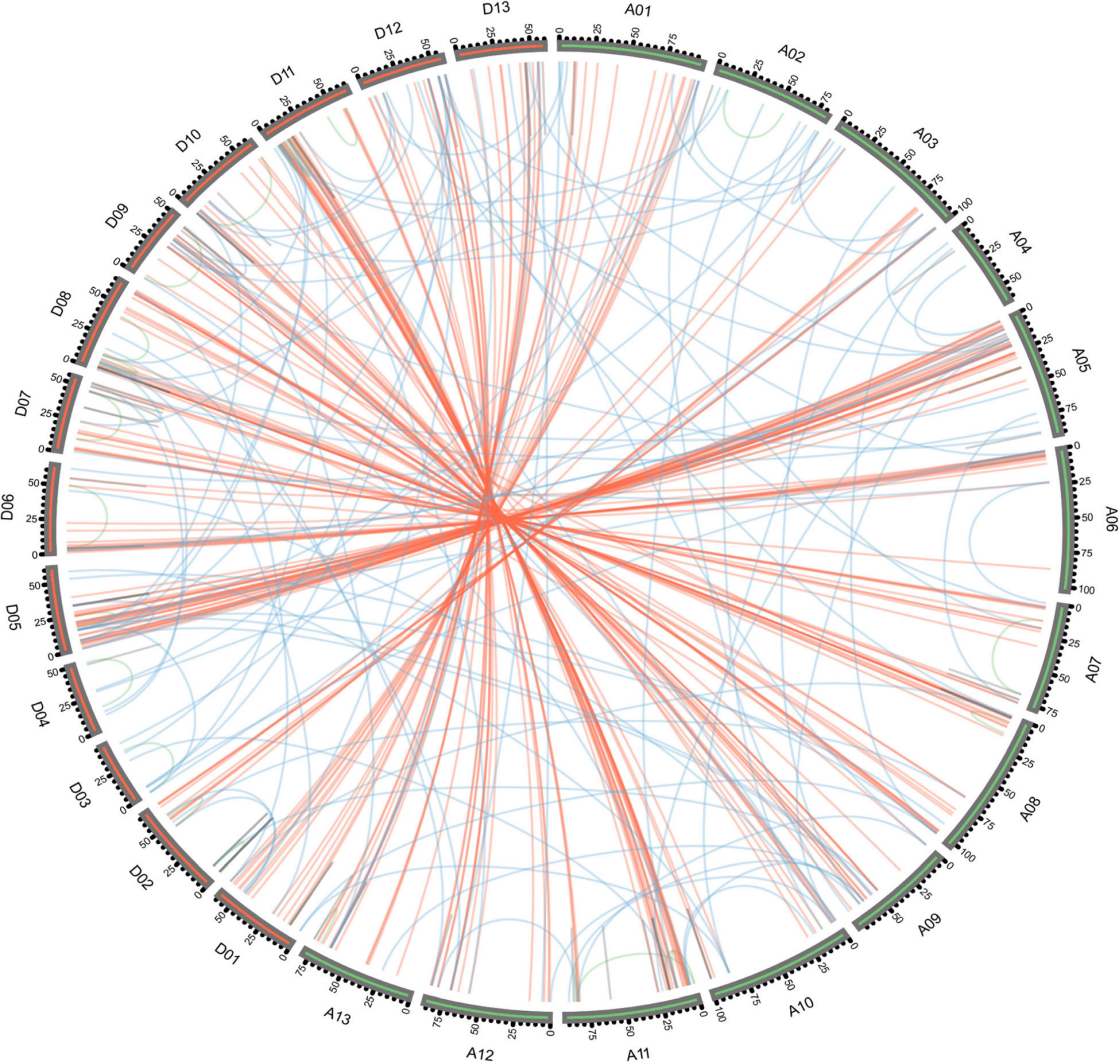
The synteny pairs of cotton F-box genes from different duplication mode diagrams. The syntenic pairs from whole genome duplication (WGD) were linked by red lines. The brown, green and blue lines represent tandem, proximal and transposed duplication F-box gene-pairs respectively

When the genome from *Gossypium arboreum* (A-genome) and the genome from *Gossypium raimondii* (D-genome) were combined to produce the allotetraploid cotton AD genome, most of the cotton genes appear to have been duplicated at the whole genome level. To elucidate the evolutionary genome rearrangement and duplication patterns of the F-box protein encoding genes in *Gossypium hirsutum*, we performed a gene duplication event analysis including whole genome duplication (WGD), tandem duplication (TD), proximal duplication (PD) and transposed duplication (TRD) (Fig. 3). A total of 303 WGD F-box genes, corresponding to 166 duplicated gene pairs, were identified in *Gossypium hirsutum* which represents the largest portion of F-box genes in allotetraploid cotton, the number of WGD duplicated genes on each of the 26 *Gossypium hirsutum* chromosomes ranging from 0 on chromosomes 4 and 17 to 22 on chromosome 5 (Additional file 1: Figure S1). 68 TD genes corresponding to 56 duplicated gene pairs, 30 PD genes corresponding to 28 duplicated gene pairs and 53 TRD, including DNA transposed duplicated and RNA transposed duplicated genes corresponding to 53 duplicated gene pairs, were also found in the *Gossypium hirsutum* F-box gene family, being distributed across 22, 13, and 16 chromosomes at low densities (Additional file 1: Figure S1). We note that the number of WGD genes is larger than that of TD, PD, and TRD genes, this finding being consistent with previous studies on the priority of modes of gene duplication in other gene families from *Gossypium hirsutum* [40, 49, 50]. The results also indicate that the F-box genes of *Gossypium hirsutum* (AD-genome) mainly originated from interspecific hybridization species *Gossypium arboreum* (A-genome) and the species *Gossypium raimondii* (D-genome).

In previous studies, major efforts were spent on identification of the contributions of WGD or TD duplications to the expansion of gene families in *Gossypium hirsutum*. In contrast, less attention was paid to the potential contributions of other modes of gene duplication such as transposed or dispersed gene duplications. As some recent studies have suggested potential roles of transposed and dispersed gene duplication to plant genome evolution [14], in the present study, we explored all possible duplication modes of the cotton F-box genes, in order to determine their potential contributions to F-box gene family expansion. We found that the order of priority of F-box gene duplication mode is WGD duplication > tandem duplication> transposed duplication >proximal duplication. This is inconsistent with previous studies in other plant species, where the duplication mode priority was found to be WGD duplication > tandem duplication > proximal duplication > transposed duplication [51–53]. Therefore, in addition to whole-genome and tandem gene duplications, other modes of gene duplication, especially transposed duplication, also contribute significantly to the evolutionary expansion of cotton F-box genes. The results from current study therefore provide further insights for understanding the mechanism of expansion of large plant gene families.

**Fig. 4:**
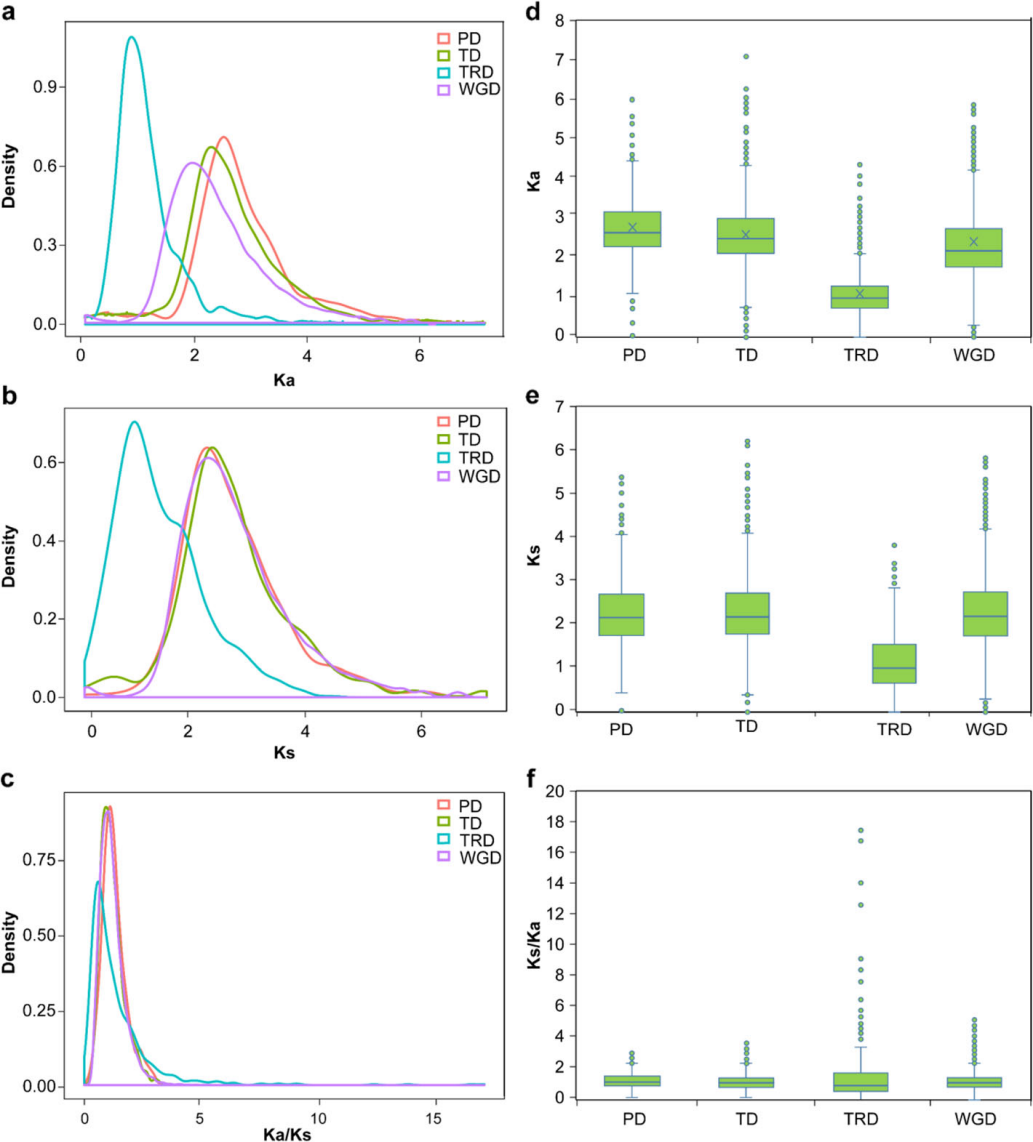
Evolutionary patterns of gene pairs duplicated by different modes in *G. hirsutum*. **a**, **d**: Ka distributions density and Box plot; **b**, **e**: Ks distributions density and Box plot; **c**, **f**: Ka/Ks distribution density and Box plot . WGD: whole-genome duplications; TD: tandem duplications; PD: proximal duplications; TRD: transposed duplications; TRD: DNA-transposed duplications

To further explore the dynamics of evolution of *Gossypium hirsutum* F-box genes, comparative studies of the different modes of gene duplication were carried out. This involved estimation of the *Ka* (non-synonymous substitutions per site), *Ks* (synonymous substitutions per site) and *Ka/Ks* ratios for each duplication pair, resulting in a measure of the divergence of cotton F-box gene family members. Without excluding extraordinarily abnormal values, we found the mean *Ka/Ks* ratio for WGD, TD, PD, and TRD were 1.2152, 1.2155, 1.302 and 1.4428, respectively (Fig. 4a-c), the mean *Ka* values for the WGD, TD, PD and TRD were 2.3404, 2.5970, 2.7078 and 1.0963, respectively, and the mean *Ks* values were 2.3404, 2.3086, 2.3126 and 1.1587, respectively (Fig. 4d-f). We calculated that the average timing of the divergence of WGD, TD, PD, and TRD mapped back to 4.5, 4.4, 4.4, and 2.2 million years ago (MYA), respectively. These results indicate that the surviving WGD, PD, and TD events had undergone a slower sequential or functional divergence for a long period. We further classified the duplicated gene pairs into three groups based on their different selection pressures (Additional file 2: Figure S2). Most of genes from different duplication modes experienced purifying selection (*Ka/Ks* > 1), which further demonstrates that cotton F-box genes have undergone positive selection during the polyploidization process*.*

### Gene ontology and organ expressions pattern of F-box protein encoding genes from *Gossypium hirsutum*

To further predict the biological functions of the F-box protein encoding genes in *Gossypium hirsutum*, Gene Ontology (GO) analysis was performed to probe orthologous genes function based on previous published F-box protein studies. Most of the cotton F-box proteins were identified as involved in the molecular function (GO:0005515) of protein binding, consistent with the established roles that F-box proteins play in post-translational modifications. In addition, 15 of the F-box genes are involved in specific biological processes (BP) (Table 1). We analyzed 15 cotton F-box protein functions further based on the known functions of orthologous *Arabidopsis* proteins [12, 14, 54]. Four genes are likely to be involved in protein ubiquitination (*Gh_A08G1127*, *Gh_A11G0844*, *Gh_D08G1412*, *Gh_D11G0986*), two genes (*Gh_D08G2231*, *Gh_A08G1869*) are involved in cell wall macromolecule catabolic processes, three genes (*Gh_D02G1602*, *Gh_D02G1375*, *Gh_D01G1559*) are involved in lateral root development, one gene (*Gh_A10G2188*) is involved in the process of response to nematode infections, one gene (*Gh_D01G1375*) is involved in flower development and two genes were assigned to no known function. In the future, it will therefore be interesting to expand the present study by exploration of their potential regulation roles in development, reproduction, and response to internal or external stimulus of *Gossypium hirsutum*.

**Table 1:** 15 F-box genes involved in biological process (BP) based on the known functions of orthologous Arabidopsis genes

Gene duplication group	Principle Transcript ID	GO Number	*Arabidopsis* ortholog genes	Putative Function of *Arabidopsis* orthologs
WGD	Gh_A08G1127	6	AT3G61590	protein ubiquitination
WGD	Gh_A11G0844	6	AT3G61590	protein ubiquitination
WGD	Gh_D08G1412	6	AT3G61590	protein ubiquitination
WGD	Gh_D11G0986	6	AT3G61590	protein ubiquitination
WGD	Gh_A05G1828	5	AT3G12350	Biological process
WGD	Gh_A13G1079	4	AT5G56180	actin filament-based process
WGD	Gh_D02G1375	4	AT3G60350	lateral root development
WGD	Gh_D07G1180	4	AT5G26960	NA
WGD	Gh_D13G1344	4	AT5G56180	actin filament-based process
WGD	Gh_A08G1869	2	AT1G55000	cell wall macromolecule catabolic process
WGD	Gh_D08G2231	2	AT1G55000	cell wall macromolecule catabolic process
PD	Gh_D02G1602	4	AT3G60350	lateral root development
TRD	Gh_D01G1559	4	AT3G60350	lateral root development
TRD	Gh_D01G1375	3	AT1G68050	positive regulation of flower development
TRD	Gh_A10G2188	8	AT3G60160	response to nematode, transmembrane transport

**Fig. 5:**
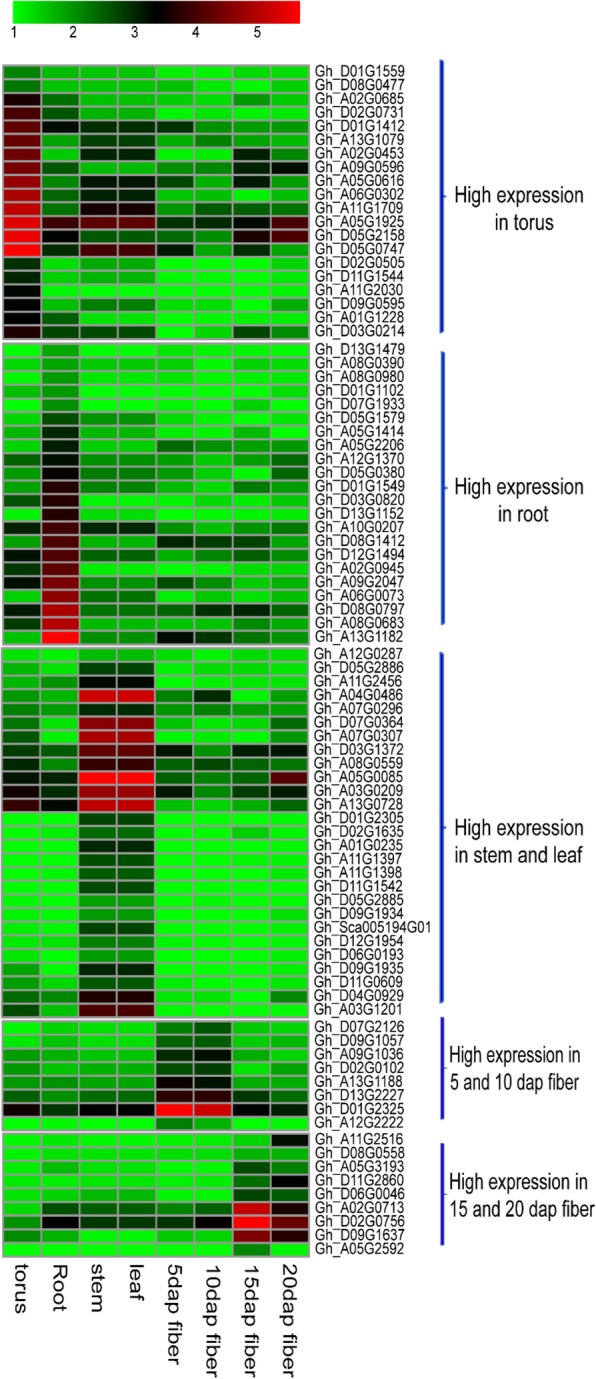
Heat map showing high expression F-box genes in 5 clusters based on K-means classification. The high expressing F-box genes in a cluster had 2 times FPKM value to other clusters. Tissue names and fiber development stages are indicated at the bottom of each lane, Color key represents log of FPKM values

The tissue-specific expression profiles of the 592 cotton F-box protein encoding genes are publicly accessible from a collection of *Gossypium hirsutum* gene expression databases. In our study, we focused on the expression profiles of cotton F-box genes in the following tissues: Leaf, Root, Stem, Torus, and fibers of different development stages (5, 10, 15, 20 dap). F-box genes having expression levels with FPKM values greater than 1 were defined as *expressed genes*. A further 440 genes with FPKM values greater than 2 and being expressed in at least one of the selected tissues (Additional file 5: Table S5) were defined as *high expression genes*. This group includes 109 genes from the torus, 91 genes from root, 106 genes from stem and leaf, 60 genes from 5 and 10 dap fibers, and 74 genes from 15 and 20 dap fibers (Additional file 3: Figure S3, Additional file 5: Table S6). K-means analysis resulted in classification of the high expression genes into 5 clusters (high expression in torus, high expression in root, high expression in stem and leaf, high expression in 5 and 10 dap fibers and high expression in 15 and 20 dap fibers) (Fig. 5 and Additional file 4: Figure S4). Among these clusters, 27 genes were found to have high expression in leaf and stem, followed by 22 in root, 20 in torus, 8 in 5 and 10 dap fibers, and 9 in 15 and 20 dap fibers. In each cluster, we noticed that several F-box genes exhibited differential expression in one or more of the cotton plant tissues. These results imply that F-box genes with high expression levels in specific tissues most likely participate in the biological processes specific to that tissue type, whereas the ubiquitously expressed F-box genes may be involved in fundamental cellular processes.

### Identification of possible F-box genes in the SCF complexes involved in plant hormone signal transduction pathways

**Fig. 6:**
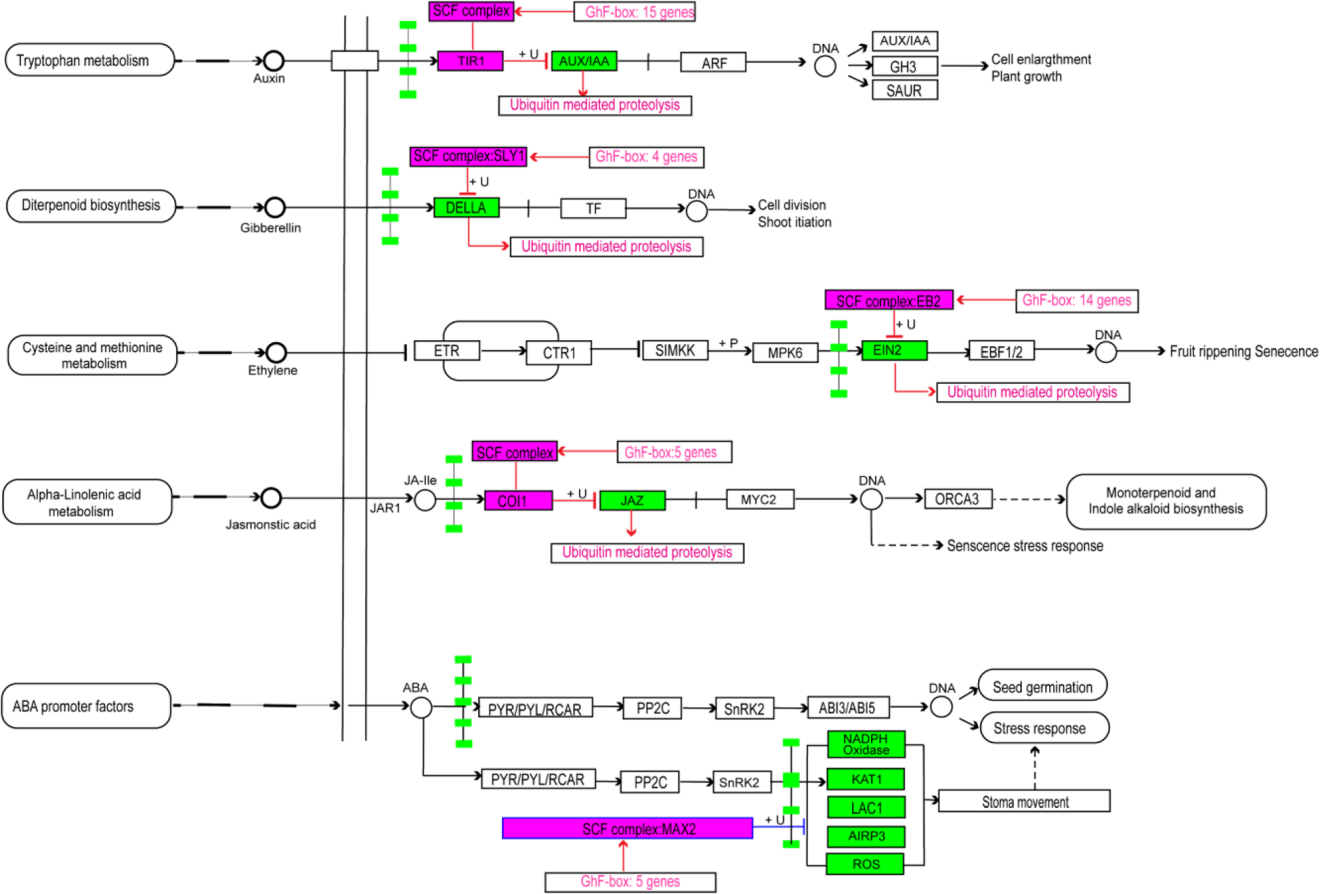
The *G. hirsutum* F-box proteins predicted to form the SCF complexes which were demonstrated involved in hormone signal transduction pathways in *Arabidopsis*. The well-established *Arabidopsis* hormone related SCF complexes were colored as pink blocks while the green color blocks represent the protein substrates or the down-stream target genes of the selected SCF ubiquitin ligases

**Fig. 7:**
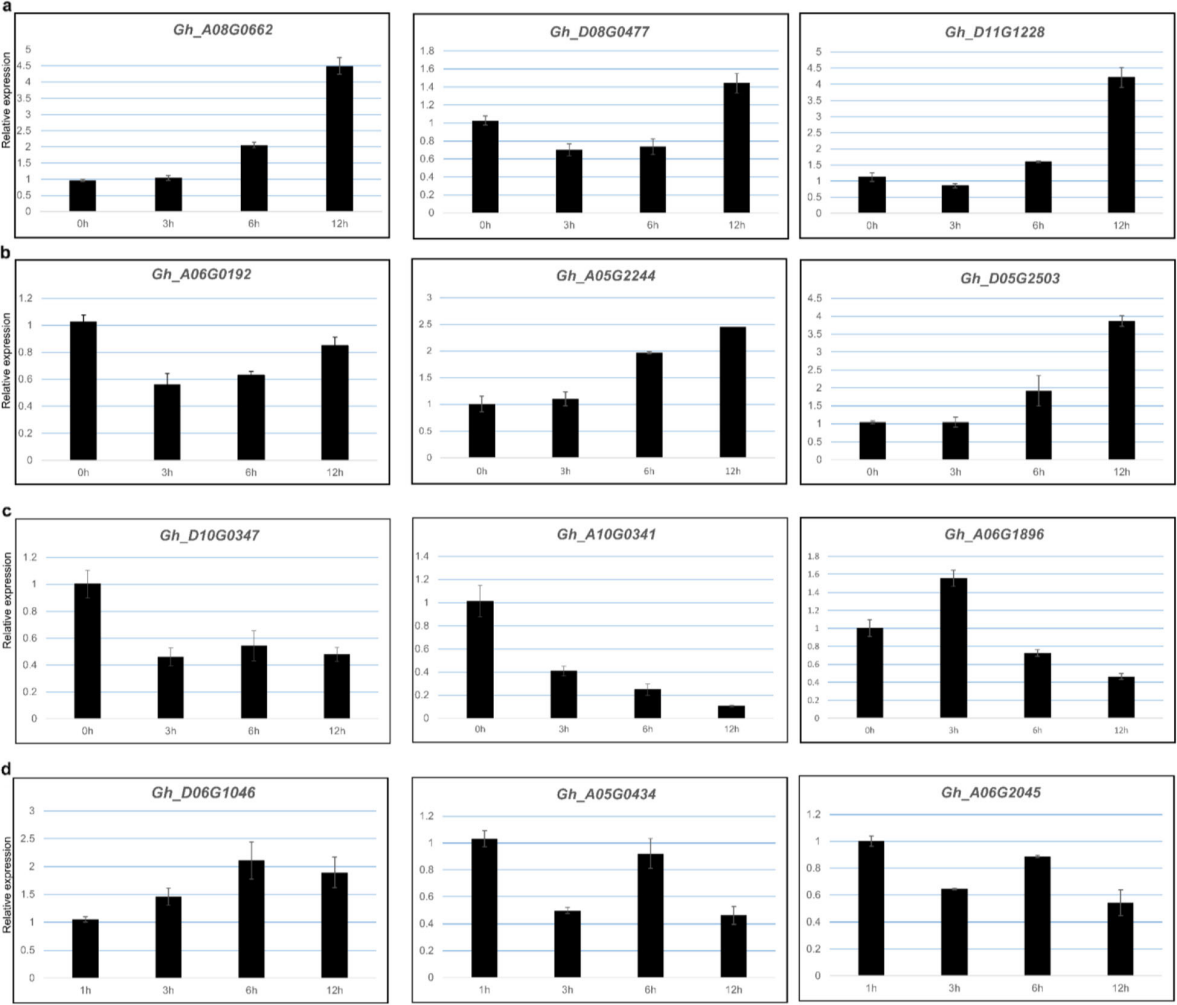
Expression dynamics of the candidate F-box genes under IBA, GA3, ABA, ACC treatments. Error bars represent SD of three independent biological experiment repeats. The value on the Y-axis indicate the relative gene expression levels. The x-axis represents the time points when the *G. hirsutum* leaf samples were collected. **a**: *TIR1* homologous genes expression changes during the course of IBA treatment; **b**: *SLY1* homologous genes expression during the course of GA3 treatment; **c**: *MAX2* homologous genes expression during the course of ABA treatment; **d**: *EB2* homologous genes expression during the course of ACC treatment

Most of the F-box proteins can form SCF complexes by binding with the Skp1 protein, and Skp1 further interacts with the scaffold proteins Cullin1 and Rbx1 to form the major components of classic SCF E3 complexes. Among all the components, F-box proteins serve as the protein recruiting components of the SCF type E3 ubiquitin ligase to determine the specificities of the substrate proteins for ubiquitination and degradation [55]. Studies performed on the model plant *Arabidopsis* reveal that the common strategy used by plants to precisely respond to hormone signals is by the modulation of the stabilities of key transcription factors by an F-box protein containing ubiquitin ligase [41]. We performed blast analysis against a *Gossypium hirsutum* cDNA library using *Arabidopsis* F-box genes as bait that have been shown to participate in the signal transduction in different hormonal pathways. 43 F-box protein encoding genes were identified as SCF complexes likely involved in different *Gossypium hirsutum* hormone signal transduction processes (Fig. 6). Among these, 15 cotton F-box proteins showed greater than 50% identity to AtTIR1 protein, the auxin receptor [56], hence likely to contribute to forming the SCF complexes that mediate the AUX/ IAA signaling process, Similarly, 4 proteins showed more than 50% identity with AtSLP1 protein, the key component of *Arabidopsis* GA signaling transduction [57], and therefore should form the SCF complexes which likely target DELLA protein for degradation by UPP to mediate gibberellin signaling. Fourteen proteins showed more than 50% identity with AtEBF2 protein, the important ethylene signaling regulator [58], and so are likely involved in SCF complexes targeting the EIN3 protein to mediate the ethylene signaling. Five COI1 homologous proteins showed more than 50% identity with AtCOI1 protein, a key regulator of jasmonate signaling [59], and therefore are likely forming the SCF complexes that mediate jasmonate signaling process by UPP. We also identified 5 proteins with more than 50% identity to the AtMAX2 protein, which plays dual roles in karrikin and strigolactone signaling [60] (Additional file 5: Table S7). Among the *AtMAX2* orthologous genes, *Gh_A12G2577* and *Gh_D12G0880* have been reported to control shoot lateral branching in cotton, consistent with their reported function in *Arabidopsis* [34]. To confirm that the expression of these potential cotton hormone responsive F-box genes is under influence of hormone homeostatic changes, quantitative real-time PCR analysis was performed. By the end of the course of IAA and GA treatments, representative AtTIR1 homologous genes (*Gh_A08G0662*, *Gh_D08G0477* and *Gh_D11G1228*), and AtSLP1 homologous genes (*Gh_A05G2244* and *Gh_D05G2503*) were all upregulated. In contrast, *Gh_A06G0192* was slightly suppressed by 12 h of GA treatment (Fig. 7). In addition, representative *AtMAX2* homologous genes (*Gh_D10G0347*, *Gh_A10G0341* and *Gh_A06G1896*) were all suppressed after 12 h of ABA treatment, which is consistent with previous report that AtMAX2 expression is suppressed by ABA treatment [42].

## Discussion

F-box family proteins are one of the super protein families in plants, and studies on a limited number of plant F-box proteins have demonstrated that they play diverse roles in various key plant development and physiological processes, including germination [18], floral meristem identity and organ development [20, 61, 62], photomorphogenesis, the circadian clock, flowering time [21, 63–66], regulation of hormone signaling transduction [41, 56–60, 67, 68], plant response to stress conditions [10, 22, 23, 42, 48, 69–76], plant nutrition usage [77], plant reproductive processes [16, 17], and plant primary and secondary metabolism [25–27, 78, 79]. Compared with the vast number of plant F-box genes that have been identified, functional characterizations of the majority of F-box proteins still lags far behind. Comprehensive genome-wide identification of plant F-box family genes is essential before the systematic characterization of their biological functions can be attempted. So far, identification of F-box protein encoding genes at the whole genome level has been reported for a number of plant species, including: *Arabidopsis* [80, 81], rice [48], grapevine [82], maize [11], apple [13], chickpea [12], *Medicago* [10], pear [14] and soybean [9]. However, information available for cotton F-box genes and for their roles in the cotton plant life cycle is limited. Here, we first conducted a genome-wide survey of the cotton F-box gene family. Subsequently, their phylogenetic relationships, gene structures, conserved motifs, chromosomal locations, duplication events and their tissue specific expression analysis via in silico analysis of publicly available RNA-sequencing (RNA-seq) database and quantitative reverse-transcription polymerase chain reaction (qRT-PCR) were employed to verify our bioinformatic predictions.

Using the well-established 1808 F-box protein sequences from *Arabidopsis*, *Populus* and rice as query sequences [8], 592 F-box protein encoding genes were identified in the new version of the *Gossypium hirsutum* genome database (http://mascotton.njau.edu.cn). When compared to other sequenced plant species, the *Gossypium hirsutum* F-box gene family is the third largest, behind rice as the largest (779 family members), and *Arabidopsis* (692 members) [8]. It is well established that *Gossypium hirsutum* genome is a result of hybridization of its two parental species *Gossypium raimondii* and *Gossypium arboretum*), and upland cotton is a classic model for plant polyploid domestication and genome scale duplication studies. Evidence also suggested that cotton polyploidization process has undergone a subtle gene loss*.* Previous studies have showed that WGD (Whole Genome Duplication) is the major driving force for the expansion of gene family members in *Gossypium hirsutum*. In addition to the analysis of contribution of WGD for cotton F-box gene expansion, we also analyzed other modes of gene duplication such as transposed or dispersed gene duplications for their potential contributions to the expansion of the cotton F-box gene family. As compared to the 592 F-box genes found in the *Gossypium hirsutum* genome, the 300 F-box genes we found in the *Gossypium raimondii* genome and the 282 F-box genes in *Gossypium arboreum* suggest that, as for other cotton gene families, the F-box gene family also experienced only subtle changes in number in term of family members after the evolution from diploid to tetraploid, and that this gene number change (a slight increase in total F-box gene number which may be the result of contributions by duplication modes other than WGD) indicates the F-box gene family members are indispensable for the enhanced traits of upland cotton and its evolutionary adaptation to variable photic environments.

The well characterized plant F-box proteins so far all contain a functional domain at their C-terminus, and the various C-terminus functional domains were found to be diverse in all the plant species studied so far, including cotton in our current study [6]. Domain analysis of the cotton F-box proteins revealed that a large portion (54%; 320 out of 592) of the predicted proteins did not have any other known functional domain beyond the F-box motif itself, and this group of F-box proteins was thereafter designated as Fbox. A similar phenomenon was also found in other plant species (Fig. 1). In the cotton genome, the Kelch domain containing F-box proteins (KFB) represent the most abundant F-box protein subfamily after Fbox (61 in total), similar to the situation in populus and maze, with 35 and 37 subfamily members, respectively (Fig. 1). In addition, *Arabidopsis* KFBs also form a third most abundant F-box subfamily proteins with about 100 members (Fig. 1) [83]. Plant KFBs have been demonstrated to be involved in a range of important biological processes including controlling photoperiodic flowering in *Arabidopsis* [21, 63], regulation of plant organ fusion and growth [20, 62, 84], controlling *Arabidopsis* clock progression [64], regulations of rice leaf senescence and yield [79], plant secondary metabolism regulation [25–27] and control of *Arabidopsis* seed germination [18]. There are no previous reports of the characterizations of cotton KFBs, and it will therefore be interesting to explore the biological functions played by cotton KFBs for their potential contributions to important agricultural traits. Consistent with other plant species, the large subfamilies of cotton F-box proteins also include FBA (46 members), FBD/LRR (34 members), LRR-repeats (9 members) and DUF (18 members) (Fig. 1). The FBA domain-containing F-box proteins have been shown to be involved in regulation of S-RNase-mediated self-incompatibility in *Arabidopsis* [85]. Almost 10% (46/592) of the cotton F-box proteins belong to FBA subfamily and, based on the established model, the functional significance of this F-box protein subfamily is also worthy to be further explored experimentally in the future.

Plant F-box proteins also have been shown to play critical roles during most of the known hormone signal transduction processes, either serving as the hormone receptors, or as key transcription regulators during hormone perception [41]. Functional characterization of most of the F-box genes so far has been limited in the model plants, such as *Arabidopsis*, and their homologues in other crop species likely play similar functions. The BLASTP algorithm-based search (E < 1e-10 and Identities > 50%) of the F-box genes carried out in our study confirmed the presence of cotton homologues of classic *Arabidopsis* hormone related F-box proteins suggesting their probable involvement in similar or identical biological pathways. For example, close homologues of *AtTIR1* [56] (*Gh_A08G0662*, *Gh_D08G0477* and *Gh_D11G1228*), *AtCOl1* [59] (*Gh_D04G0642*, *Gh_Sca006609G01* and *Gh_A05G2749*), *AtSLY1* [86] (*Gh_A06G0192*, *Gh_A05G2244* and *Gh_D05G2503*), *AtMAX2* [42] (*Gh_D10G0347*, *Gh_A10G0341* and *Gh_A06G1896*) and *AtEB2* (*Gh_D06G1046*, *Gh_A06G2045* and *Gh_A05G0434*) which are known to be involved in plant growth and development upon the homeostasis changes of auxin, JA, GA, ABA, and ethylene were readily identified in the cotton F-box gene family (Fig. 6). To investigate the potential functions of these homologues in cotton plants responding to major hormone signaling changes, we performed quantitative RT PCR analysis of the expressions of the representative genes after the cotton leaves were treated with IAA, JA, GA, ABA, and ACC. By the end of IAA treatment, all three *AtTIR1* homolog genes expression were significantly up-regulated comparing to the control plants (Fig. 7a). This finding is consistent with the previous published role of AtTIR1 protein as the auxin receptor in *Arabidopsis* [87]. Although, there is scant evidence showing that auxin can affect *AtTIR1* gene transcript levels, our data clearly showed that auxin regulation of plant growth and development is through both transcriptional and post-translational modifications. Jasmonate and related signaling compounds are not only playing the important roles during the processes of plant responses to both biotic and abiotic stresses, but also are crucial for plant growth and development. JAZ1 protein is the key transcription repressor of jasmonate-responsive genes and is degraded by the COI1 containing SCF complex upon JA treatment [59]. Five *AtCOI1* homologs were detected in our analyses. GA is another important plant hormone which has been demonstrated to modulate *Arabidopsis* plant growth and development through the regulation of levels of phosphorylated DELLA protein, the repressors of GA responsive genes, achieved by rapid induction of DELLA protein degradation by SCF^SLY1^ complex [57]. Thus, similar to AtTIR1 and AtCOI1, AtSLY1 is the crucial component of *Arabidopsis* GA signaling transduction module. We analyzed the potential effects of GA treatment on the expression of the 4 cotton homologues of *Arabidopsis* SLY1. Two (*Gh_A05G2244* and *Gh_D05G2503*) of the three selected genes showed steady increases over the course of GA treatment. However, *Gh_A06G0192* displayed suppressed expression at all the sampling points (Fig. 7c). These data suggest that Gh_A06G0192 may confer its function in a opposing fashion as compared to the remaining two F-box proteins. Further protein-protein interaction experiments will be needed to verify whether any of these SLY1 homologues can interact with the cotton DELLA protein, in order to directly determine whether they are serving as the F-box protein component of the cotton SCFS^LY1^ complex participating in the cotton GA signaling pathway. The *Arabidopsis* F-box protein MORE AXILLARY GROWTH2 (MAX2) has previously been characterized for its role in plant branching and MAX2 appears essential for the perception of the newly-characterized phytohormone strigolactone, a negative regulator of polar auxin transport in *Arabidopsis*. In addition, MAX2 has also been shown to be involved in karrikin signaling. But the detailed molecular mechanism awaits to be elucidated [60]. A number of studies also have indicated that MAX2 contributes to plant responses to both biotic and abiotic stresses through modulation of the ABA signaling pathway and that *MAX2* gene expression can be influenced by ABA treatment [42, 75, 88]. Bu et al showed that *MAX2* expression was suppressed to about 50% after Arabidopsis seedlings were treated with 50 μM ABA for 6 h [42]. After performing a BLAST search, we identified 5 cotton homologues of *MAX2*, and three of these were selected for modulation of gene expression in response to ABA treatment. Consistent with previous reports, the three cotton *MAX2* genes are all suppressed by ABA, especially *Gh_A10G0341* expression which steadily decreases during the ABA treatment. The protein substrate for AtMAX2 is not known in *Arabidopsis*, but considering its important role during plant growth and development (branching would be a particularly beneficial trait directly related the yield of cotton and other crop plant species), it will be interesting to further investigate the functions of cotton *MAX2* genes in the future.

## Conclusions

Cotton is the most important crop for renewable fiber production. In this study, a systematic genome-wide analysis of F-box gene family was performed for the first time as the complement to a number of recent cotton genome sequencing projects. As a result, 592 F-box genes were identified and subjected for further structural and phylogenetic characterizations. Our analysis led to the identification of a number of conserved F-box subfamilies present in the cotton genome which show close similarity at the amino acid level to other model and crop plant species. Gene duplication event analysis showed that, from diploid cotton (A-genome parent and D-genome parent) to the allotetraploid AD-hybrid, the expansion of the cotton F-box gene family is exclusively achieved by whole genome duplications (WGD) with only subtle contributions from other gene duplication modes. Digital expression profiles of the F-box genes across different tissues were also explored and several F-box genes were identified with tissue specific expression patterns implying their possible involvement in the growth and development of selected organs. A combination of homology searches, classic hormone treatments and RT-PCR experiments, identified putative F-box genes likely to be involved in cotton hormone signaling transduction pathways. This study serves as a foundation for the selection and characterization of candidate genes to be used for trait improvement in cotton breeding programs.

## Supplementary information

**Additional file 1: Figure S1.** The expansion modes of F-box genes in upland cotton. a: The number of F-box genes of different duplication modes. The x-axis represents gene numbers, and the Y-axis represents different duplication modes; b: Distribution of duplicated genes on 26 chromosomes of upland cotton. The x-axis represents chromosome numbers, and the Y-axis represents the gene number of different duplication modes.

**Additional file 2: Figure S2.** The proportion of duplicated gene pairs under different (evolution) selection forces. Red dots represent purifying selection where the Ka/Ks value is smaller than “1”; Green dots represent neutral selection where the Ka/Ks equals to “1”; Blue dots represent positive selection where the Ka/Ks is bigger than “1”. Abbreviations: WGD (whole-genome duplication); TD (tandem duplication); PD (proximal duplication); TRD (DNA-transposed duplication).

**Additional file 3: Figure S3.** Organ specific expression of F-box genes in the *G. hirsutum*. Color scale represents log transformed RPKM values. Light green indicates low expression and red color indicates high expression. Heatmap was generated using R program.

**Additional file 4: Figure S4.** The optimal number of clusters (K) as determined by ‘k-means’ clustering. The red line indicated the optimal number of clusters is at five.

**Additional file 5: Table S1.** Detailed information of FBOX members from Upland cotton. **Table S2.** Detailed information of F-box members from G. raimondii. **Table S3.** Detailed information of F-box members from G.arboreum. **Table S4.** Upland cotton F-box gene numbers for different modes of gene duplication. **Table S5.** Organ-specific expression patterns of F-box genes. **Table S6.** Optimal numbers of clusters (K) as determined by ‘k-means’ clustering. **Table S7.** F-box gene as the SCF complexes involved in hormone signal transduction based on the homologous gene in Arabidopsis. **Table S8.** real-time PCR primers.

## Data Availability

The data sets supporting the results of this article are included within the article and its additional files.

## Abbreviations

BP: biological process
CP: cellular component
GO: Gene Ontology
MF: molecular function
PD: proximal duplications
RPKM: reads per kilobase per million
TD: tandem duplications
TRD: transposed duplications
WGD: whole-genome duplications
