# Supplementary figures and images for: Genome-wide analysis and characterization of F-box gene family in Gossypium hirsutum L
Source: BMC Genomics. 2019 Dec 19;20:993. doi: 10.1186/s12864-019-6280-2 (PMC6921459; doi:10.1186/s12864-019-6280-2)

## Slide 1
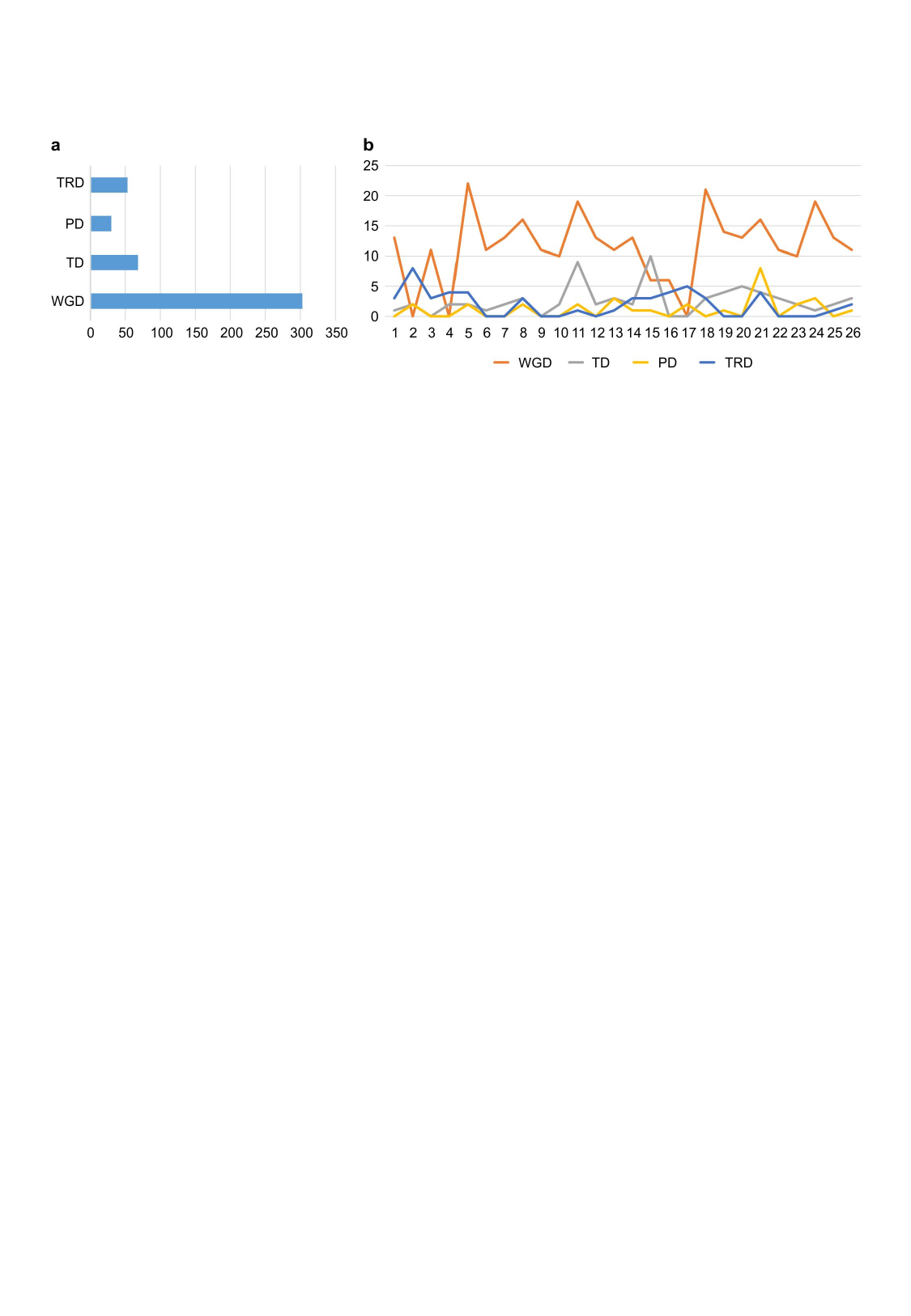

Supplement: Supplementary file 1 — Additional file 1: Figure S1. The expansion modes of F-box genes in upland cotton. a: The number of F-box genes of different duplication modes. The x-axis represents gene numbers, and the Y-axis represents different duplication modes; b: Distribution of duplicated genes on 26 chromosomes of upland cotton. The x-axis represents chromosome numbers, and the Y-axis represents the gene number of different duplication modes. [file 12864_2019_6280_MOESM1_ESM.pptx]

## Slide 1
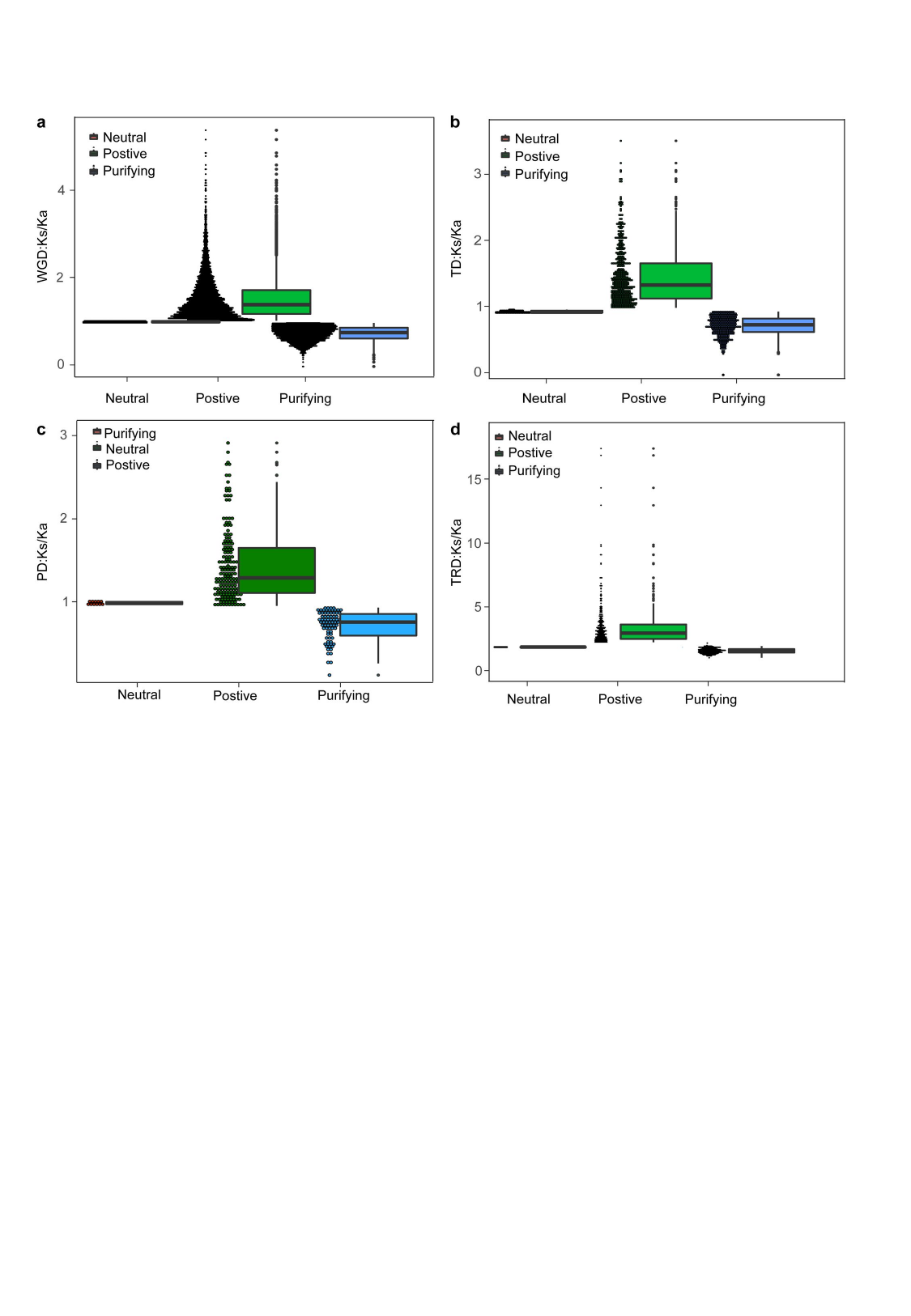

Supplement: Supplementary file 2 — Additional file 2: Figure S2. The proportion of duplicated gene pairs under different (evolution) selection forces. Red dots represent purifying selection where the Ka/Ks value is smaller than “1”; Green dots represent neutral selection where the Ka/Ks equals to “1”; Blue dots represent positive selection where the Ka/Ks is bigger than “1”. Abbreviations: WGD (whole-genome duplication); TD (tandem duplication); PD (proximal duplication); TRD (DNA-transposed duplication). [file 12864_2019_6280_MOESM2_ESM.pptx]

## Slide 1
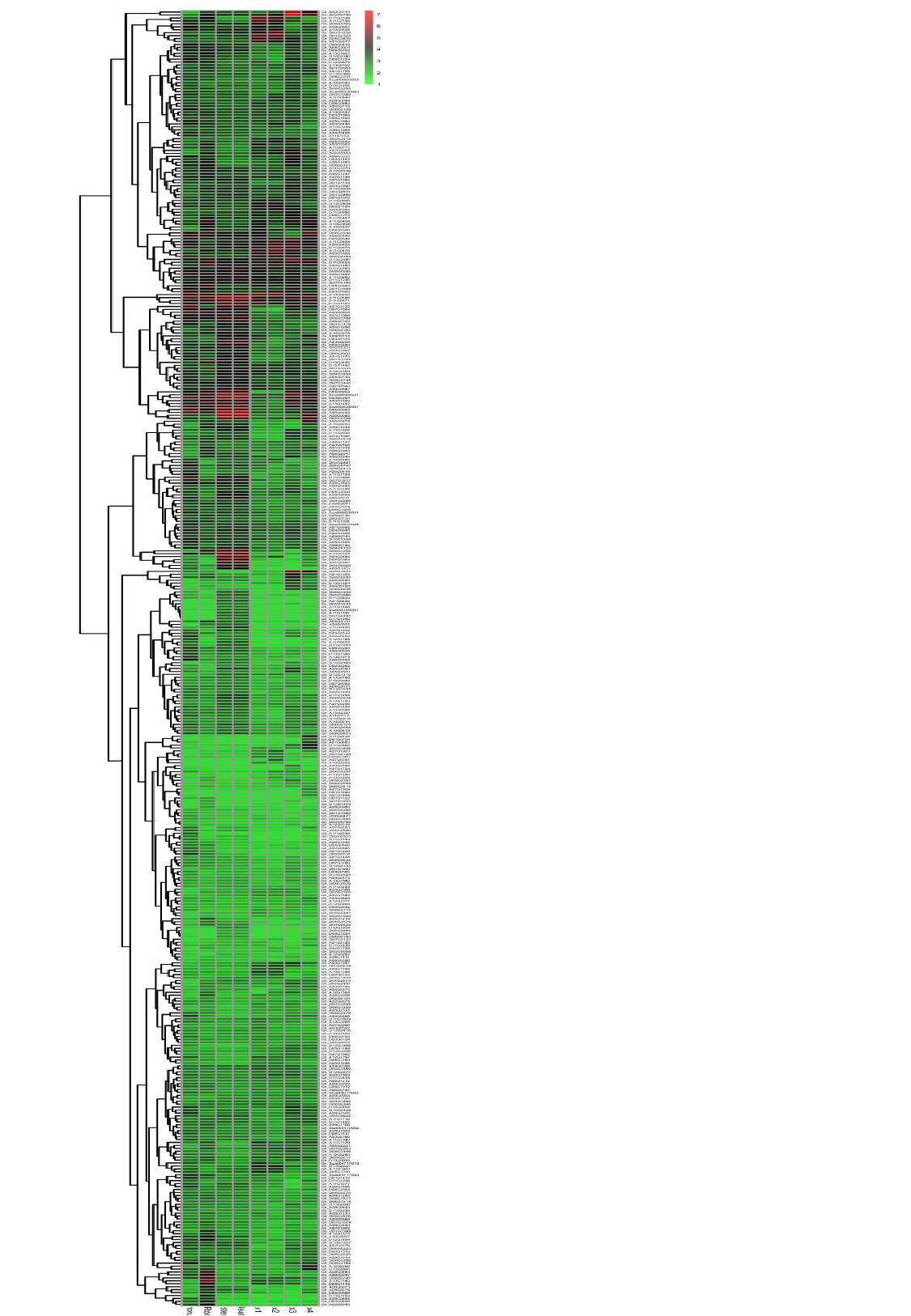

Supplement: Supplementary file 3 — Additional file 3: Figure S3. Organ specific expression of F-box genes in the G. hirsutum. Color scale represents log transformed RPKM values. Light green indicates low expression and red color indicates high expression. Heatmap was generated using R program. [file 12864_2019_6280_MOESM3_ESM.pptx]

## Slide 1
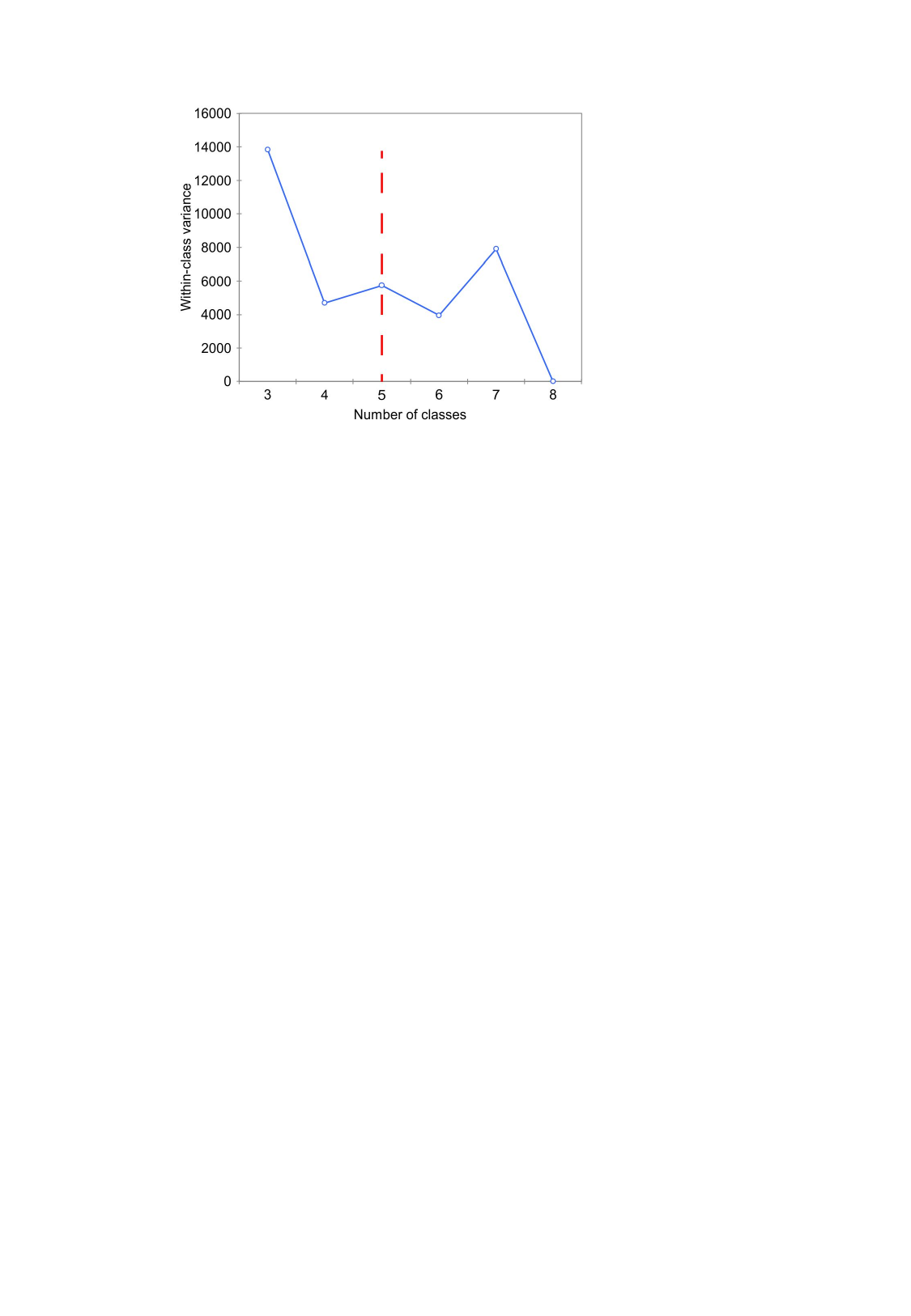

Supplement: Supplementary file 4 — Additional file 4: Figure S4. The optimal number of clusters (K) as determined by ‘k-means’ clustering. The red line indicated the optimal number of clusters is at five. [file 12864_2019_6280_MOESM4_ESM.pptx]
